# Supplementary material for: Augmenting the human interactome for disease prediction through gene networks inferred from human cell atlas
Source: Anim Cells Syst (Seoul). 2025 Mar 7;29(1):11–20. doi: 10.1080/19768354.2025.2472002 (PMC11892045; doi:10.1080/19768354.2025.2472002)
Supplement: Supplementary_Figure1 [file TACS_A_2472002_SM7900.pdf]

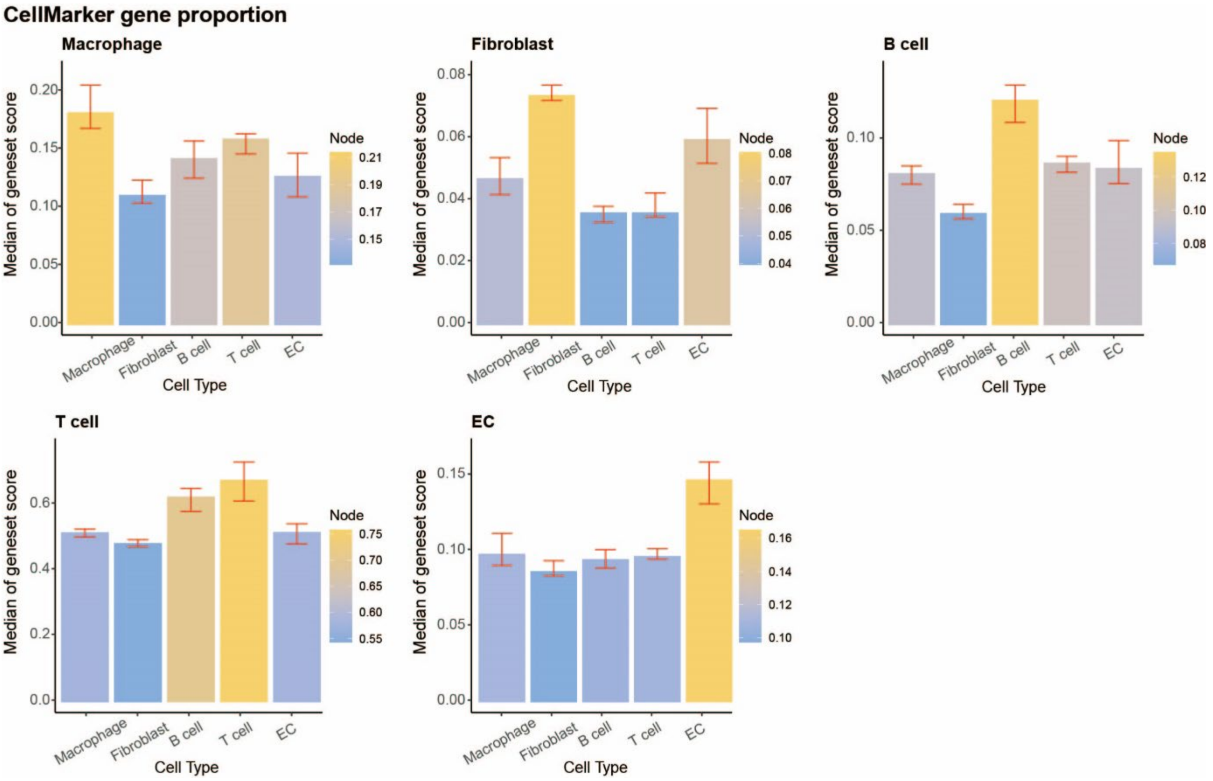

Supplementary Figure 1. Proportion of cell type-specific marker genes in the corresponding cell-type-specific network. The bar graphs represent the median proportion across four cancer types (breast, colorectal, lung, and ovarian cancers), with error bars indicating the range of scores across these cancer types.
